# Intelligent Inter- and Intra-Row Early Weed Detection in Commercial Maize Crops

**DOI:** 10.3390/plants14060881

**Published:** 2025-03-11

**Authors:** Adrià Gómez, Hugo Moreno, Dionisio Andújar

**Affiliations:** 1 Laboratorio de Propiedades Físicas: Técnicas Avanzadas en Agroalimentación LPF-TAGRALIA, School of Agricultural, Food and Biosystems Engineering (ETSIAAB), Technical University of Madrid, Avenida Puerta de Hierro 2-4, 28040 Madrid, Madrid, Spain; 2 Centre for Automation and Robotics, CSIC-UPM, Ctra. M300 Campo Real, Km 0,200, 28500 Arganda del Rey, Madrid, Spain; hugo.moreno@csic.es

**Keywords:** deep learning, object detection, site-specific weed management (SSWM), intra-row weeding, visual transformer, maize, energy efficiency

## Abstract

Weed competition in inter- and intra-row zones presents a substantial challenge to crop productivity, with intra-row weeds posing a particularly severe threat. Their proximity to crops and higher occlusion rates increase their negative impact on yields. This study examines the efficacy of advanced deep learning architectures—namely, Faster R-CNN, RT-DETR, and YOLOv11—in the accurate identification of weeds and crops within commercial maize fields. A comprehensive dataset was compiled under varied field conditions, focusing on three major weed species: *Cyperus rotundus* L., *Echinochloa crus-galli* L., and *Solanum nigrum* L. YOLOv11 demonstrated superior performance among the evaluated models, achieving a mean average precision (mAP) of 97.5% while operating in real-time at 34 frames per second (FPS). Faster R-CNN and RT-DETR models achieved a mAP of 91.9% and 97.2%, respectively, with processing capabilities of 11 and 27 FPS. Subsequent hardware evaluations identified YOLOv11m as the most viable solution for field deployment, demonstrating high precision with a mAP of 94.4% and lower energy consumption. The findings emphasize the feasibility of employing these advanced models for efficient inter- and intra-row weed management, particularly for early-stage weed detection with minimal crop interference. This study underscores the potential of integrating State-of-the-Art deep learning technologies into agricultural machinery to enhance weed control, reduce operational costs, and promote sustainable farming practices.

## 1. Introduction

Weeds have long been a major challenge to agricultural production, as they compete with crops for essential resources such as sunlight, water, nutrients, and space, ultimately diminishing crop yield and quality [1]. For instance, weeds can cause potential yield reductions of approximately 44% in the USA and Canada [2] and 20–30% in India [3]. Inadequate weed management can result in yield losses of up to 90% [4]. Moreover, the rapid growth and spread of weeds can trigger pest and disease outbreaks, often leading to fungal infections [5]. Therefore, managing weeds is vital for ensuring agricultural productivity. In a crop field, the inter-row zone is the area between adjacent rows of plants, defined by the spacing left to allow for machinery movement, irrigation, or other field operations. Furthermore, the intra-row zone refers to the space between individual plants within the same row, which is determined by the planting density and arrangement. Together, these zones define the spatial organization of the crop.

Weeds in the intra-row zone are especially problematic, potentially reducing yields by 18% to 76%, which underscores the importance of their eradication [6]. Weed removal in the intra-row zone is more difficult than in the inter-row zone due to the closer proximity of weeds to the crop line [7]. Effectively controlling weeds in the intra-row zone without harming crops presents a significant challenge. The core difficulty is ensuring weed removal while preserving the integrity of the crops [8]. Inter-row weeding can be performed without disturbing the crop row, whereas intra-row weeding demands real-time crop avoidance to prevent damage [9]. However, manual weed control methods are time-consuming and have low effectiveness, typically removing only 65–85% of the weeds on average [10]. This emphasizes the importance of integrating automated intra-row weeding to enhance sustainable land use and reduce reliance on human labor [11].

Various systems with no real-time weed and crop location have been proposed due to their reduced complexity and costs. Chandel et al. [6] developed a tractor-drawn integrated inter- and intra-row weeding system for field crops, using active rotary tines for intra-row weeding and passive tines for inter-row. The system achieved a weed mortality of 92.8% in maize and 84.1% in pigeon pea crops. Trajkovski et al. [12] proposed a simple modular lightweight e-hoe for inter- and intra-row weeding, accomplishing the task with 95% of weeds removed. However, these methods have shown inefficiency in speed and accuracy when used in different fields, even though they were explicitly designed for a particular crop field [13].

Site-specific weed management (SSWM) adapts treatment strategies to the specific density, distribution, and types of weeds in an area. By optimizing weed control interventions, this method reduces herbicide usage, cuts production costs, and lessens environmental impact. Thus, effective SSWM relies on accurately identifying the precise location of the weeds to be treated and distinguishing the weed species when necessary [14]. This information is essential for implementing intelligent weed management strategies that optimize treatment precision and efficacy. With advances in computing power and camera technology, computer vision has shown significant potential for the rapid classification and localization of crops and weeds [15]. In particular, deep learning (DL) techniques are highly effective at extracting intricate patterns from large datasets, making them a significant breakthrough in image classification. When used for crop and weed classification, DL methods have improved accuracy and overall results [16]. This improved performance enhances the integration into automated and robotic systems for efficient real-time weed management. Recent research has exploited DL techniques for crop and weed identification and localization, delivering exceptional results in automated weeding. Visentin et al. [17] introduced a semi-autonomous robotic weeding system featuring a fully integrated three-axis platform and a vision system mounted on a mobile rover. Spatial, color, and depth information acquired using an RGB-D camera was processed to classify crops and weeds through PlantNet, identifying the plants above 95%. Quan et al. [9] employed a detection model (YOLOv3) for crop and weed identification integrated into a mobile robot platform with two intelligent weeding units employing weed control knives. The system successfully removed 85.91% of weeds while maintaining a low crop injury rate of 1.17%. Jiang et al. [18] developed an intra-row weeding system based on an improved YOLOv5 detection model and open/close weeding knives. The enhanced model detected crops and weeds with 95% precision, while the system successfully removed the intra-row weeds with 80.25% accuracy at 3.28 km/h. Similarly, Hu et al. [19] improved a YOLOv7 model to identify the crops and weeds, resulting in a new lightweight Multimodule-YOLOv7-L model reporting a 97.5% accuracy in plant identification. Zheng et al. [8] designed and developed an electric swing-type opening and closing intra-row weeding control system. The system integrated a YOLOv5 model for accurate identification and localization of cabbage, enabling dynamic obstacle avoidance for the weeding knives, reaching a weeding accuracy of 96.00%, with a crop injury rate of 1.57% at 0.1 m/s in field experiments. In contrast, other authors focused on techniques to improve weed detection in general without focusing specifically on inter- or intra-row detection. López-Correa et al. [20] trained three detection architectures to identify seven species in tomato crops. They trained YOLOv7, RetinaNet, and Faster R-CNN performing at around 92% mAP. Differently, Hugo et al. [21] focused on increasing and studying the performance of DL models, training them through artificial images generated through Stable Diffusion techniques. YOLOv8 and RetinaNet were first trained using only real images, and their performance was compared with subsequent training conducted with artificial images combined with real ones. The performance improvement reported was around 8% and 9% in both models.

Most existing research focuses on improving either recognition performance or recognition speed, with few studies addressing both issues simultaneously [22]. The difficulties of managing intra-row weeds, where the proximity to crops increases the risk of crop damage, add complexity to the management process, as does the requirement to simultaneously increase speed and precision [7]. To address these challenges, this study proposed utilizing diverse, State-of-the-Art neural networks, enabling fast and accurate inter- and intra-row area detection. Our research aimed to evaluate the performance of Faster R-CNN, RT-DETR, and YOLOv11 detection models trained and optimized to detect inter- and intra-row weeds in commercial maize fields. The target weeds included two monocotyledonous species (*Cyperus rotundus* L. and *Echinochloa crus-galli* L.) and one dicotyledonous species (*Solanum nigrum* L.). Additionally, the hardware performance of the detection models was analyzed, including various configurations of the best-performing model. The research aimed to advance knowledge in developing high-precision CNNs to ensure precise and efficient maize and weed detection, hence minimizing crop damage. Furthermore, energy efficiency was assessed to confirm each model’s suitability for integration into precision weed control systems.

## 2. Materials and Methods

### 2.1. Image Collection

**Figure 1:**
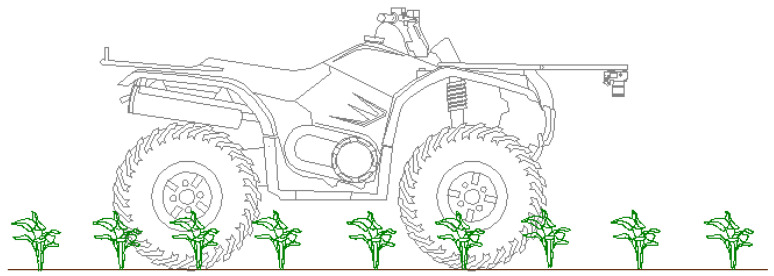
Schematic positioning of the camera mounted on an ATV.

The selected fields for the experiments were commercial maize fields located in the province of Badajoz, Spain (39°1′14.42″–6°3′40.69″). The commercial fields were sampled at the coordinates 39°01′11.7″ N 6°02′40.2″ W; 38°59′49.2″ N 6°03′23.0″ W; 38°58′46.6″ N 6°02′23.9″ W. Images were collected under natural, uncontrolled illumination conditions over several days and at various times, capturing diverse soil backgrounds, shadows, and lighting variations. Both maize crops and weeds were included in the scenes, with weeds present along crop lines and interlines. A significant portion of the weeds in crop lines overlapped with maize plants, particularly during advanced growth stages. The images were captured during the early stages of maize and weed development, coinciding with the period of weed treatment. No herbicide spraying was conducted during the image acquisition or the preceding 10 days. Each field lot was surveyed using an “M” trajectory, with zenithal images taken every 2 m from a height of 1.3 m. Two monocotyledonous weed species (*Cyperus rotundus* L. and *Echinochloa crus-galli* L.) and one dicotyledonous weed species (*Solanum nigrum* L.) were documented in this study. All were in low growth stages and observed alongside maize crops (*Zea mays* L.) from V1 to V4 growth stages [23], corresponding to early developmental phases. The images were captured in April 2023 using a Canon PowerShot SX540 HS camera with a 5184 × 3886 pixel resolution (Tokyo, Japan). The camera was mounted on agricultural machinery (an ATV) to facilitate a standardized and quantifiable data collection method, ensuring constant height (Figure 1). The camera was configured with a shutter speed of 1/1000, and the ISO was automatically calibrated to adjust to changing lighting conditions. This setup ensured high-quality images representing the interactions between weeds and maize crops under varying environmental and lighting conditions.

### 2.2. Corn and Weed Dataset Construction

The image annotation process was conducted manually by weed science experts using LabelImg software (version 1.8.6), a free and open-source graphical image annotation tool developed in a Python environment. LabelImg was used to define the locations of weed instances by creating bounding boxes around the weeds. The annotations were saved in the PascalVOC format (XML), allowing the export of the coordinates for each labeled weed. Each image’s bounding boxes were stored collectively in a single XML file, with each object including the coordinates of its upper-left and lower-right corners (xmin, ymin, xmax, and ymax) along with its class name. The five prominent weed species identified were classified based on the EPPO (European and Mediterranean Plant Protection Organization) code system. A total of 4388 images were labeled, generating 54,368 bounding boxes. In this research, we used 70% of the data for training, 10% of the data for validation, and 20% for testing. Table 1 shows the instances in each class used for training, validating, and testing the models.

**Figure 2:**
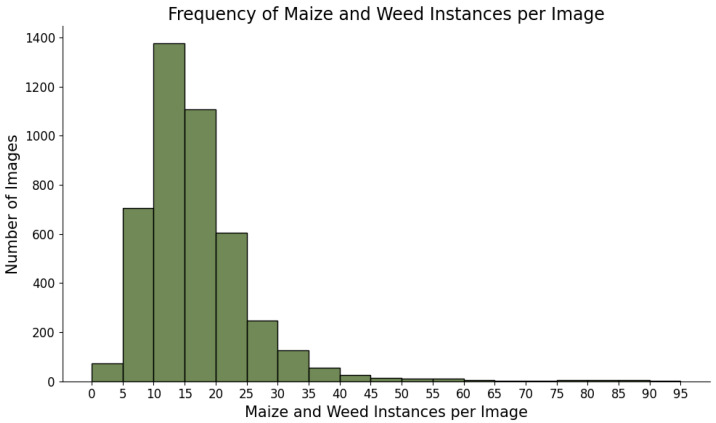
Distribution of the number of maize and weed instances detected per image across the dataset. The *x*-axis represents the range of maize and weed instances per image. The *y*-axis measures the frequency, indicating the number of images that fall within each instance range.

The labeled weeds were mostly identified in the embryonic or early stages of development. However, there were also developed weeds, although they were a minority. Each image contained around 16 labels on average. Most images (2651 images) contained between 10 and 20 instances, 781 images of the total contained less than 10 instances, and 956 images contained more than 20 instances. Among the 956 images containing more than 20 instances, images with more than 80 instances were found. Figure 2 shows the frequency of instances per image in the complete dataset. In addition, the images contain mostly weeds in the early stages of development, giving bounding boxes of reduced sizes, which implies a major challenge for the detection models. The original images, measuring 1296 pixels in height and 3000 pixels in width, were considered unmanageable due to the small size of the weeds, the large image dimensions, and the GPU’s processing constraints. The images were resized by segmenting each into two smaller sub-images to tackle this issue. This process was guided by three parameters: the height and width of the sub-images and the overlap between them. The chosen dimensions were 1296 pixels for height, 1650 pixels for width, and 300 pixels for overlap. Correspondingly, the XML files containing plant labels were adjusted to align with the segmented images, producing a separate XML file for each sub-image. During segmentation, bounding boxes at the edges of sub-images were often truncated, resulting in incomplete data. These truncated bounding boxes were excluded from the training dataset to maintain data integrity. The specified overlap ensured that any plants excluded due to edge truncation reappeared in neighboring sub-images, thereby preserving all weed seedlings from the original images within the training set.

**Figure 3:**
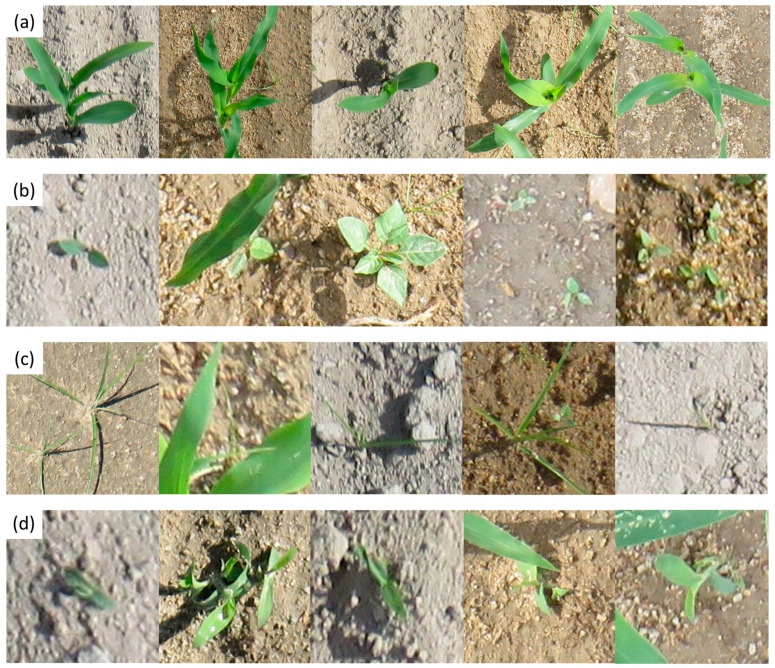
Representative samples of captured variations in plant development and environmental conditions, i.e., lighting variations, occlusions, and different background colors and textures of the dataset by classes. (**a**) *Zea mays* L., (**b**) *Solanum nigrum* L., (**c**) *Cyperus rotundus* L., (**d**) *Echinochloa crus-galli* L.

In conclusion, the dataset utilized in this study encompasses images capturing the early to mid-growth stages of both weeds and maize, providing comprehensive coverage of critical developmental phases. This ensures that the trained model can accurately detect and distinguish between plant species at various points in their growth cycles, from initial emergence to more established stages. Additionally, the images were collected under diverse lighting conditions, including natural sunlight at different times of the day and varying levels of cloud cover. The dataset also incorporates multiple soil backgrounds, such as dark, loamy soil, lighter sandy textures, and soils with varying moisture levels, as shown in Figure 3. This diversity in environmental factors enhances the models’ robustness and generalization capability, allowing it to perform effectively across different field conditions and improving its adaptability for real-world agricultural applications.

### 2.3. Experimental Setup

**Figure 4:**
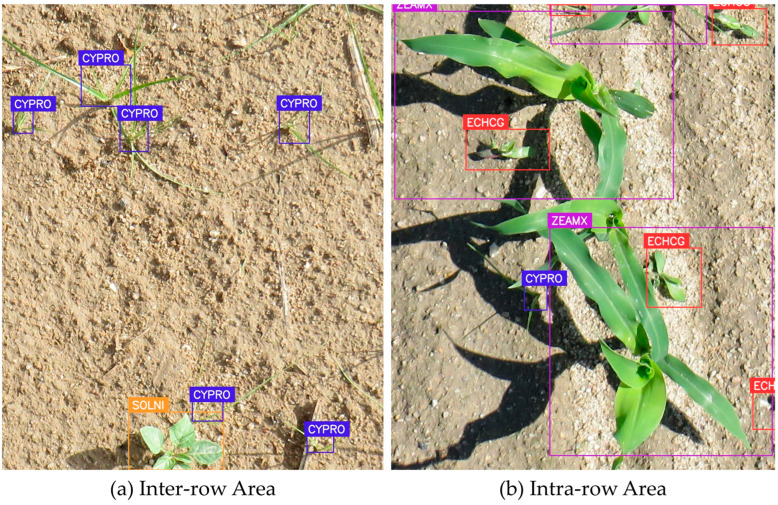
Examples of images used to assess the detection models’ performance. Image (**a**) shows an intra-row section of the crop field with CYPRO and SOLNI weed species, while image (**b**) shows an intra-row section with the weed species ECHCG and CYPRO between and overlapping with the maize (ZEAMX) plants.

The collected images contained both inter-row and intra-row zones. The intra-row zone is challenging for weed detection due to the close proximity of weeds to crops and complex occlusions [18]. Developing a combined system for inter-row and intra-row weeding could save time, power, and costs compared to performing them as separate processes [6,24]. A single detection model was proposed to detect weeds and crops in both zones at the same time (inter- and intra-row). Therefore, different detection architectures were trained and tested with inter- and intra-row images. Figure 4 shows a sample of an image collected inter- and intra-row. The different detection models were essentially tested on intra-row images. However, some of these intra-row images contained part of the inter-row area alongside. DL models require extensive datasets to accurately capture the features of objects and enhance their generalization and robustness across varied scenarios [25].

**Figure 5:**
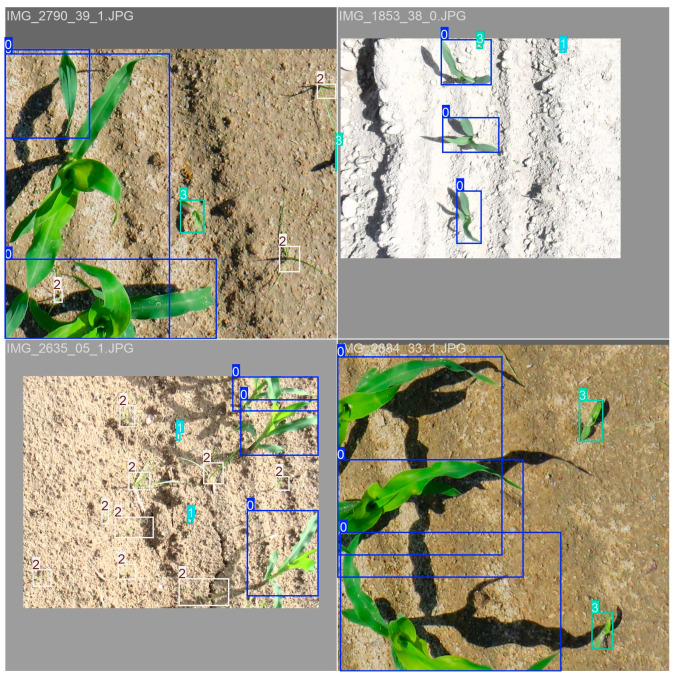
Image samples used in the training process with data augmentation applied to the images.

Data augmentation, as outlined by Maharana et al. [26], was applied to significantly expand the dataset size and diversity to address the limitations of a small dataset. This process utilized several augmentation techniques, including zooming, shearing, horizontal flipping, brightness modification, cropping, image rotation, and the addition of noise. These transformations generated new, varied samples from the original data while preserving critical features, thereby simulating a range of real-world conditions. These modifications were performed in real-time at the same instant as the training, allowing a different and random modification to be applied to each image at every training epoch. This approach reduced memory as saving all the newly modified images was unnecessary, although the training was slightly decelerated. This strategy expanded the dataset, providing the model with a richer and more diverse set of training examples to improve its performance and adaptability. Zooming was applied within a range of [0.8, 1.2], permitting the model to learn from magnified and reduced weeds’ perspectives. Shearing transformations with an angle of up to 10 degrees were introduced, creating geometric alterations that mimicked the natural variability in the shapes and orientations of weeds. Horizontal flipping generated mirrored images, ensuring the model could recognize weeds regardless of their directional orientation. Brightness adjustments, ranging from −15% to +15%, simulated varying lighting conditions to increase adaptability. Random cropping within pixel intervals of (0, 10) was used to replicate scenarios with partially obscured weeds or varying fields of view. Rotations of up to 10 degrees exposed weeds viewed from multiple angles, reflecting real-world positional variations. Minor translations were incorporated to reposition weeds and crops across different areas of the image. Additionally, noise was added to up to 0.1% of the image pixels to simulate sensor imperfections and variability in real-world data. Together, these augmentation strategies expanded the diversity of the dataset, improving the models’ capability to identify weeds accurately across a wide range of environmental and imaging conditions. Figure 5 shows samples of augmented images used in the training process.

In addition to testing the models’ precision, each model’s hardware performance was analyzed. Therefore, prediction time, inference time in frames per second, model size, memory required for model execution, number of model parameters, and FLOPS were measured. Considering the performance of the models based on the accuracy of the predictions and the hardware, the different sizes of the best-performing architecture were trained and analyzed. Thus, different sizes of the same architecture were examined to determine how they affect intra- and inter-row detection of weeds and crops. This study not only evaluated the performance of different models in detecting weeds in intra- and inter-row environments within maize fields but also analyzed the optimal size of the best-performing architecture for deployment in real-time applications.

### 2.4. Selected Object Detection Models

Among the AI techniques utilized in precision weeding, DL has been highlighted as a key method due to its ability to model and learn complex patterns in data using multi-layered artificial neural networks. DL techniques, particularly Convolutional Neural Networks (CNNs), are employed to process visual data effectively, identifying weeds by analyzing spatial patterns in images, which helps in applying treatments precisely where needed [27]. Object detection models, a type of CNN, employ bounding boxes to facilitate the identification and localization of multiple weed instances within an image [28]. These models are especially useful because they can detect image patterns automatically and effectively. In this study, three different architectures of object detectors were employed to detect the intra- and inter-row weeds of maize crops. Faster R-CNN, RT-DETR, and YOLOv11 were the selected architectures to perform the detection. The object detection models utilized pre-trained weights based on the large-scale COCO dataset. These pre-trained weights provided the models with an initial proficiency in object detection, leading to transfer learning that saves computational time.

#### 2.4.1. Faster R-CNN

Faster R-CNN [29,30] is a two-stage, region proposal-based object detection model that enhances its predecessors (R-CNN and Fast R-CNN) by incorporating a fully convolutional Region Proposal Network (RPN). Faster R-CNN operates in two main stages. The first stage involves the network components: the backbone, feature pyramid, and region proposal network, which together extract and propose candidate regions for object detection. The second stage focuses on the region of interest (RoI) alignment, followed by classification and bounding box regression, which refine the proposed regions and produce the final detection results. The ResNeXt-101-FPN backbone was employed in this study, leveraging pre-trained models to utilize the architecture and the benefits of transfer learning.

#### 2.4.2. RT-DETR

Real-Time Detection Transformer (RT-DETR) is a cutting-edge object detection framework that balances real-time performance with accuracy. Extending DETR’s NMS-free approach, RT-DETR integrates a convolutional backbone and a hybrid encoder to achieve efficient processing. Its flexible design allows users to adjust inference speed by varying decoder layers without retraining. The model’s advancements include an Efficient Hybrid Encoder, which processes multi-scale features by separating intra-scale interactions and cross-scale fusion for improved efficiency; IoU-Aware Query Selection, which focuses on the most relevant objects to enhance detection precision; and Adaptable Inference Speed, which enables real-time adjustments in speed through scalable decoder configurations.

Optimized for accelerated environments like CUDA with TensorRT, RT-DETR delivers superior real-time detection capabilities compared to other models [31].

#### 2.4.3. YOLOv11

You Only Look Once (YOLO) was introduced in 2016 [32]. YOLO revolutionized object detection by treating it as a regression problem rather than performing separate classifications for each region. This innovative approach has led to multiple iterations, from YOLOv1 to the latest YOLOv11 in 2024. YOLOv11 [33] represents the latest evolution in the YOLO series of real-time object detectors, setting new benchmarks for accuracy, speed, and efficiency. This iteration introduces an enhanced backbone and neck architecture for superior feature extraction, introducing the C2PSA and the C3k2 modules, enabling more precise object detection and better handling of complex tasks. YOLOv11 also incorporates refined architectural designs and optimized training pipelines, ensuring faster processing speeds while maintaining an excellent balance between accuracy and performance. Designed for adaptability, YOLOv11 seamlessly integrates across diverse environments, including edge devices, cloud platforms, and NVIDIA GPU-supported systems. Furthermore, it supports a broad range of computer vision tasks, such as object detection, instance segmentation, image classification, pose estimation, and oriented object detection (OBB), making it a versatile tool for tackling varied challenges.

### 2.5. Training Environment

Training an object detection model involves extensive computations and significant memory usage, resulting in high hardware requirements. Therefore, all experiments were performed on a node equipped with an Intel^®^ Xeon^®^ Gold 6240R processor (Santa Clara, CA, USA) and an NVIDIA A100 GPU (Santa Clara, CA, USA). All processes were implemented using Python 3.11. The detection models were trained with PyTorch 2.5.1 and CUDA 12.1, harnessing GPU capabilities to accelerate model training. For inference, the detection models were exported to the TensorRT format, optimizing NVIDIA GPUs to achieve maximum speed in frame detection. During the experimental procedures, to ensure a fair comparison across the different networks, consistent parameters were maintained for batch size, learning rate, training epochs, and initial weights for all networks under evaluation (Table 2).

## 3. Results

In this study, training and performance analysis of models was conducted in two different sets. The first set involved training, analyzing, and comparing the performance of the Faster R-CNN, RT-DETR, and YOLOv11 detection models. The largest size of each model was selected. Specifically, Faster R-CNN ResNeXt-101-FPN, RT-DETR-X, and YOLOv11x were used. On the other hand, the second set comprised training and analyzing the performance of the different sizes of the best-performing detection model of the first set. To ensure a qualitative precision evaluation, the used test dataset contains a wide variety of images. Hence, the test dataset included images of weeds and maize from early to mid-growth stages, with dense vegetation, as shown in Figure 2. Moreover, a stratified split was applied to ensure that class distribution was preserved across all sets; hence, the dataset set contained diverse lighting conditions, including varying times of day, cloud cover, and multiple soil backgrounds, such as dark loamy soil, light sandy textures, and soils with different moisture levels. Also, to ensure that the test set accounted for occlusions, plants partially obscured by leaves, shadows, or overlapping vegetation were included as part of the entire dataset (Figure 3).

### 3.1. Analysis of Maize and Weed Detection

Faster R-CNN, RT-DETR, and YOLOv11 were trained and tested, with the data split shown in Table 1. The detection precision of every model was evaluated using the individual class average precision and the mean average precision (mAP) to calculate the overall model performance. After the training and evaluation process, all the models showed good performance and efficacy for weed and maize detection in the inter- and intra-row environments. The precisions achieved for each model are listed in Table 3. All models exceeded 0.9 mAP, demonstrating that all models achieved proficient and satisfactory accuracies adequate for their application. Faster R-CNN obtained the lowest result with 0.919 mAP. This architecture found ZEAMX to be the most difficult class to detect, with a precision of 0.868, while the rest of the classes all exceeded 0.91. Despite the competent result, Faster R-CNN lags behind the results reported by RT-DETR and YOLOv11, which obtained mAPs of 0.972 and 0.975, respectively. These high accuracies show the strong potential of these models in the detection of maize and weeds in inter- and intra-row environments. ZEAMX was again the most challenging class to detect for both models, although it exceeded 0.94 accuracy in this case. RT-DETR and YOLOv11 were capable of identifying maize with an accuracy of about 9% greater than Faster R-CNN. CYPRO was the second class with the largest gap between these models. Faster R-CNN obtained about 6% less precision than RT-DETR and YOLOv11 detecting CYPRO. The SOLNI and ECHCG classes reported the highest accuracy for all models. RT-DETR and YOLOv11 reached 0.98 precision, while Faster R-CNN lagged by 4–5%. RT-DETR and YOLOv11 obtained closely comparable results, varying by less than 1% in the accuracies attained for every class. This is reflected in the reported mAPs, where the difference between both models is marginal. Difficulties in the detection of ZEAMX demonstrate the complexity of the intra-row zone detection due to possible occlusions with weeds and even occlusions between different corn plants. ZEAMX was the largest species observed in this study, characterized by its long and thin leaves. This morphological feature results in bounding boxes covering a large area, often including significant portions of the background and leaves from neighboring crops or nearby weeds. Such extensive background inclusion can interfere with model training by giving undue importance to non-crop elements, thereby complicating the accurate detection of the crop itself. Despite this challenge, the reported accuracies indicate that RT-DETR and YOLOv11 outperformed other models, proving the most effective for detecting weeds and crops in inter-row and intra-row zones.

**Figure 6:**
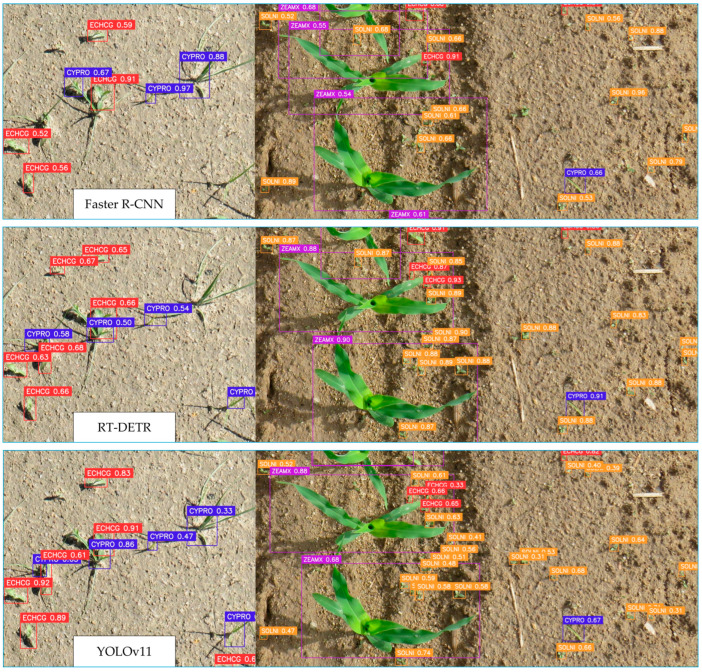
Samples of the detection results obtained for each detection model. The purple bounding boxes correspond to the ZEAMX species, the orange to SOLNI, the blue to CYPRO, and the red to ECHCG classes.

Figure 6 presents a comparative visual evaluation of Faster R-CNN, RT-DETR, and YOLOv11 applied to weed and crop detection. The left section of each row represents detection results in a scenario with sparse vegetation on bare soil, while the right section illustrates performance in a denser crop environment. Faster R-CNN demonstrated precise localization and classification, especially for large, well-defined objects. However, it struggled with detecting smaller weeds, becoming the model with most misclassifications. RT-DETR successfully identified a greater number of weed instances compared to Faster R-CNN, achieving detections close to YOLOv11, though some misclassifications occur in dense vegetation areas. The YOLOv11 model detected the highest number of weed instances, particularly in challenging environments with occlusions, yet was very similar in performance to RT-DETR.

### 3.2. Analysis of the Optimal Object Detection Model

Precision is not the only measure to consider when comparing different detection architectures. The hardware performance of each model is also a crucial aspect to be analyzed. To measure the hardware performance, the following values were analyzed: (i) the number of parameters, (ii) the model disk size, (iii) the quantity of VRAM needed for training, (iv) the average time required to complete a training epoch, (v) the floating-point operations per second (FLOPS), reflecting how many floating-point arithmetic operations the model needs to perform to process an input showing the energetic efficiency of the model, (vi) the inference time per frame, and (vii) the number of frames that can be processed in one second. The values reported for each detection architecture are listed in Table 4.

RT-DETR is the largest model, featuring 67.3 million parameters and requiring the most storage at 133.03 MB. On the other hand, YOLOv11 is the smallest, with 56.9 million parameters and taking up 114.01 MB of disk space. However, this size difference is relatively insignificant, given that modern hardware can easily accommodate all three models. Although YOLOv11 was the model with the lowest number of parameters, it required the highest amount of VRAM for training (30.4 GB), followed by RT-DETR with 29.2 GB and Faster R-CNN with 24.7 GB. However, decreasing the training batch and image sizes may reduce the required VRAM. A major difference was found in the FLOPS. This metric is crucial as it shows the necessary number of operations to process an input and, therefore, illustrates the required power to process the input frame in inference and training operations. Faster R-CNN lagged far behind the other models in efficiency, requiring more than three times the number of operations per second of YOLOv11, with 594 G FLOPS and 195.5 G FLOPS, respectively. RT-DETR achieved similar efficiency to YOLOv11, requiring 36.9 more operations per second. Yet YOLOv11 proved to be the most efficient model energetically due to its lower FLOPS requirements. The inference time is critical in a real-time system since it determines the number of frames per second that can be processed, considering that this time must be combined with the interaction time of the actuators and other system components. Regarding the inference time required to process each frame, Faster R-CNN still lagged. Faster R-CNN needs 90.37 ms, on average, to process every frame, while RT-DETR and YOLOv11 need 35.92 ms and 28.91 ms, respectively. The difference was again over three times greater between Faster R-CNN and YOLOv11, reflected in the number of frames per second that could be processed: 11 for Faster R-CNN and 34 for YOLOv11. The RT-DETR inference time was proficient, enabling 27 frames per second to be processed. However, YOLOv11 managed to process seven frames per second more than RT-DETR.

**Figure 7:**
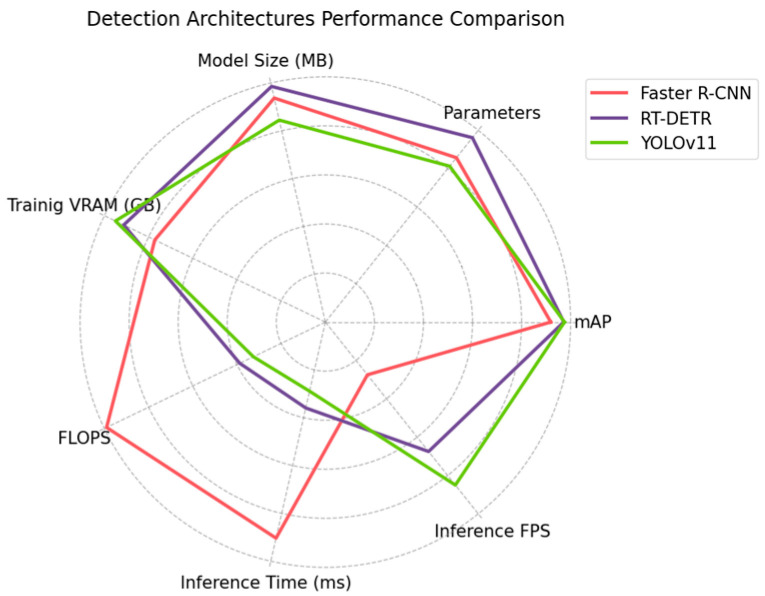
Performance comparison of Faster R-CNN, RT-DETR, and YOLOv11 in terms of precision and efficiency, highlighting each model’s strengths and trade-offs in detection accuracy, processing speed, and overall effectiveness.

Figure 7 shows a comparison between the models according to their accuracy and efficiency. The difference in efficiency between Faster R-CNN and the other models was significant. Although the precision gap was not extremely large, the difference in efficiency placed Faster R-CNN far behind the rest of the models for its application. RT-DETR and YOLOv11 did not show any particularly significant differences, yet YOLOv11 showed better values in most metrics. The analyzed results suggest that Faster R-CNN is the weakest model to be employed in a real environment, as its hardware performance is significantly lower. It is also the architecture that reported the lowest precision. The hardware performance differences between RT-DETR and YOLOv11 were not huge; however, YOLOv11 reported better values for all metrics except for the amount of VRAM required. Combined with the minimal precision difference between the two models, this performance positioned YOLOv11 as the most advantageous model for being employed in real-time applications.

### 3.3. Analysis of the Optimal Model Size

Within the same detection architecture, there are generally different model sizes. Larger sizes imply higher detection precision but also more resource consumption. On the other hand, a smaller size results in lower detection precision but reduced resource consumption. The resources of field weed management systems are not unlimited, and trying to save on the costs of these systems is critical. Therefore, finding the size that best suits the needs of the task is crucial, as well as providing a model that works efficiently and with sufficient precision. After training, analyzing, and comparing the different detection architectures, YOLOv11 was the model with the best metrics for deployment. Thus, the different available sizes of YOLOv11 were trained and analyzed, and their hardware performances were extracted. These sizes were YOLOv11x, YOLOv11l, YOLOv11m, YOLOv11s, and YOLOv11n.

After training and evaluating the models, reported precisions differed by no more than 11% between the best model (YOLOv11x) and the worst model (YOLOv11n). Table 5 shows the results obtained for the different sizes of YOLOv11. The results shown by the models ranged from better to worse, aligned with the YOLOv11 sizes. YOLOv11l showed a 2% drop in performance compared to YOLOv11x (the best-performing model). This drop in performance was produced primarily in the detection of the ZEAMX class, which decreased from 0.952 to 0.916 precision. In contrast, the rest of the species remained above 0.95. On the other hand, the variance measured between YOLOv11l and YOLOv11m was just 1%, reporting mAPs of 0.954 and 0.944, respectively. This minor drop in performance was again concentrated in the ZEAMX class, with all other species remaining nearly constant. YOLOv11s obtained a mAP of 0.914, representing a 3% decrease with respect to YOLOv11m. The most affected classes were CYPRO and SOLNI, which reported a 3.5% and 4.1% reduction in precision. ZEAMX reached 0.871 precision, while ECHCG remained at 0.95, the class with the lowest precision decay. Finally, YOLOv11n reported the largest drop in performance among the models. YOLOv11n reached 0.867 mAP, with SOLNI being the most affected class, dropping from 0.929 to 0.842 in precision compared to YOLOv11s. CYPRO and ECHCG also reported significant performance declines around 4% and 5%, respectively, while ZEAMX remained almost constant with a difference of less than 1%. These results show how model size impacts accuracy, with YOLOv11x, YOLOv11l, and YOLOv11m emerging as the most promising models to employ.

YOLOv11x demonstrated the highest detection accuracy among the evaluated sizes; however, its hardware requirements may be excessively demanding. Consequently, hardware performance metrics were analyzed for all YOLOv11 model sizes. Table 6 shows the comparison of the reported values. The difference in the number of parameters between the models is highly significant. YOLOv11x has twice the number of parameters as YOLOv11l, which is reflected in the disk size of each model, which is also double. The difference between YOLOv11l and YOLOv11m was considerably smaller, with about 5 million parameters, with disk sizes of 53.8 MB and 42.82 MB, respectively. YOLOv11s and YOLOv11n were the models with the smallest disk size, occupying 22.59 MB and 9.10 MB, respectively, with just 2.6 million parameters for YOLOv11n. YOLOv11n occupied more than 12 times less disk memory than YOLOv11x. The VRAM needed to train the models can be increased or decreased by varying the batch size and the size of the images. With the values selected for training, YOLOv11x needed 30 GB of VRAM, which implies a GPU designed specifically for model training that may be excessively expensive. YOLOv11l needed 20 GB of VRAM, though 10 GB less than YOLOv11x; this was a high volume that required a high-quality GPU. YOLOv11m required 15.8 GB of VRAM, an amount that is possible to run on some more conventional GPUs. YOLOv11s and YOLOv11n consumed a large amount of VRAM that is executable on many conventional computers, which implies greater accessibility to these models at a lower cost. However, these values can be greatly reduced by decreasing the batch size and image resolution, allowing larger models to be used on less expensive hardware. FLOPS have a direct impact on the models’ energy consumption. YOLOv11l needed to perform half the floating point operations per input (87.3 G FLOPS) compared to YOLOv11x (195.5 G FLOPS). This difference considerably reduces the energy required to process each image. The difference between YOLOv11m and YOLOv11l was 20 G FLOPS, providing a small reduction in power requirements. YOLOv11s was a very efficient model requiring three times fewer operations than YOLOv11m, although YOLOv11n was the most efficient model, performing 6.4 G FLOPS per image. YOLOv11n has the advantage that it can be run on most hardware as its power requirements are very low. However, YOLOv11s also had optimal requirements for many systems.

Regarding inference times, all models were able to process video at 30 FPS, which is generally the standard at present. However, the time required for this inference must be added to the delays of the actuators and the various mechanical components of the systems used in the field. These delays may cause a model capable of processing 34 FPS, such as YOLOv11x, to process no more than 30 FPS. YOLOv11l was able to process 40 FPS while YOLOv11m reached 47 FPS. The smaller models processed 54 FPS (YOLOv11s) and 58 FPS (YOLOv11n). In addition, a model capable of processing more FPS than required by the task increases energy efficiency by not requiring the model to be constantly executing. These metrics suggest the use of the smallest possible models as they ensure energy and cost efficiency. Even so, the precision that each size was capable of providing should not be ignored.

**Figure 8:**
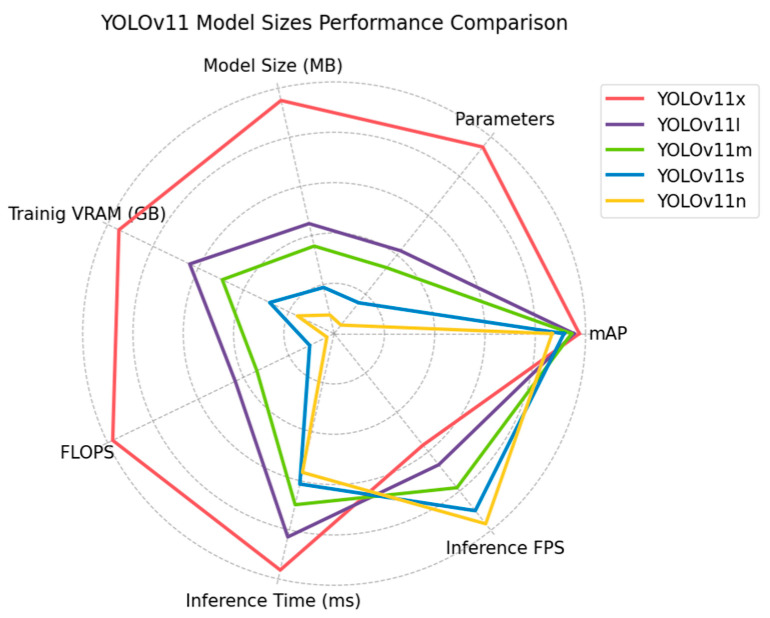
Comparative performance analysis of YOLOv11x, YOLOv11l, YOLOv11m, YOLOv11s, and YOLOv11n based on precision and efficiency, highlighting key differences in detection accuracy, processing speed, and overall effectiveness, showcasing each model’s strengths and limitations.

Figure 8 presents a comparative analysis of the models based on their accuracy and efficiency. The difference in efficiency between YOLOv11x and the rest of the models was significant. The model obtained the best mAP in exchange for a considerably lower efficiency. The performance difference between the remaining models was not so large. YOLOv11l and YOLOv11m performed similarly, with YOLOv11m being more efficient for a small loss in accuracy. YOLOv11s and YOLOv11n were clearly the most efficient models. YOLOv11s sacrificed part of the mAP in exchange for a significant reduction in FPS and an increase in inference speed. YOLOv11n, while being the best model in efficiency, its reduction in precision was very significant. Even so, it is a very interesting model for systems with limited computational capabilities. Considering the performance of each model in terms of hardware and accuracy achieved, the most optimal sizes for utilization and deployment in real systems were YOLOv11m and YOLOv11s. They were the sizes with the best trade-off between efficiency and precision. Depending on the power needs of the system and the required accuracy, one size will be more suitable than the other. YOLOv11s had very low energy requirements while being able to achieve a mAP above 0.91. However, with a system capable of executing a larger model, YOLOv11m achieved a mAP of 0.944, ensuring better weed management. In case system cost and energy efficiency are not an issue, YOLOv11x obtained excellent performances, reaching 0.975 mAP and ensuring outstanding weed treatment.

**Figure 9:**
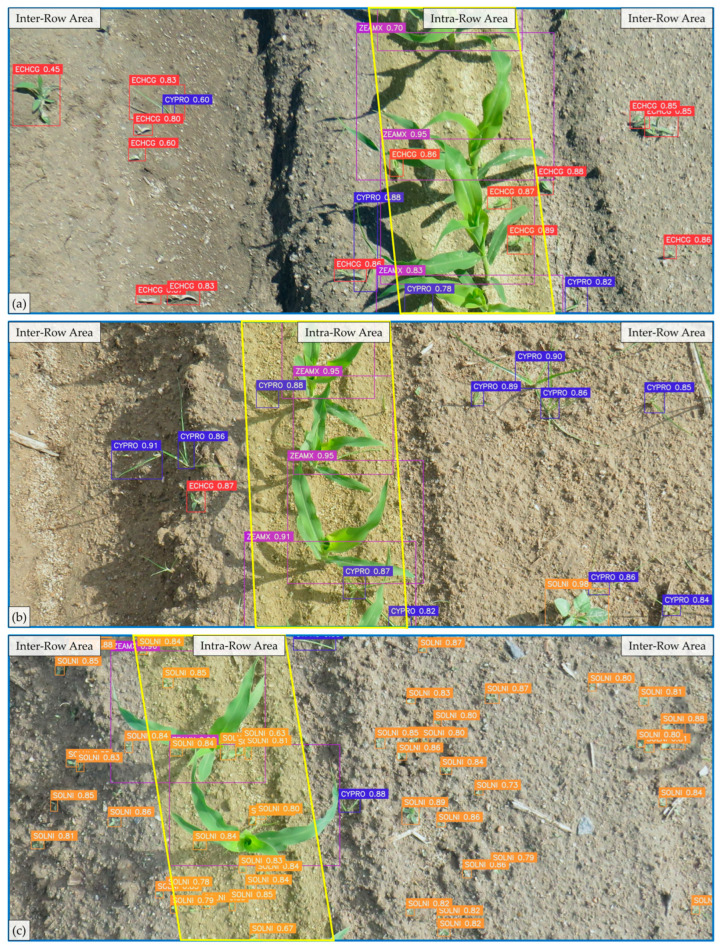
Visualization of the detections made by YOLOv11m in both inter-row and intra-row areas of a field. The intra-row area, delineated by a yellow highlight, measures approximately 280 mm in width. The remaining space in the image represents the inter-row areas. Examples of these inter-row and intra-row areas are shown in (**a**–**c**).

Based on a thorough comparison of all models, YOLOv11m was selected to process inter- and intra-row images to analyze its performance in simulating a real environment. Figure 9 shows the detections performed through YOLOv11m. The figure shows the intra-row area in yellow (280 mm width), while the rest of the image corresponds to the inter-row area. The inter- and intra-row areas were defined as indicated in Chandel et al. [6]. YOLOv11m efficiently detected weeds and maize in the inter- and intra-row areas. Image (a) shows how the model effectively detected CYPRO and ECHCG close to the crop in the intra-row area. The inter-row area in image (b) was less populated, but the CYPRO found there was correctly identified. In this image, one CYPRO weed in the inter-row area was not detected, showing how the model may contain some errors in detection. In both images, the maize overlapped due to the growth stage and the proximity between the different plants. This complicates the detection and identification of the bounding box for each instance. Even so, the model successfully detected and separated the maize in both images. Image (c) shows SOLNI in the early stage of development, in addition to numerous instances in the intra-row zone. However, YOLOv11m correctly detected all instances in the image. Moreover, in this image, numerous SOLNI were located close to the crop, and occlusions were efficiently detected. Figure 9 shows the effectiveness of the YOLOv11 model in detecting weeds and maize in the inter- and intra-row zones, demonstrating promising potential applications in both areas.

## 4. Discussion

The application of DL in automated weed control has been significantly impeded by various factors, such as the complexity of field environments and the limited capabilities of computers integrated into weeding machinery [34]. The intra-row zone is very sensitive as weeds grow between crop plants and find themselves extremely proximate to them [18]. This zone usually displays a major complexity due to the intensified occlusions between crops and weeds and possible occlusions between weeds. Therefore, accurate detection of weeds in intra-row areas is essential for correct treatment without damaging the crop [9]. Moreover, rapid and accurate detection is crucial for real-time detection. Currently, inter-row and intra-row weeding are performed as separate operations, requiring significant power and time while often incurring high costs [35,36]. A combined all-in-one inter- and intra-row weeding system would serve as a next-generation alternative [6,24]. Due to the necessity of rapid and precise predictions, researchers have conducted studies and proposed methods to solve these issues with remarkable achievements.

YOLO algorithms have been widely employed for this purpose in earlier studies [17,18,37]. Quan et al. [9] developed an intelligent intra-row weeding robot platform based on YOLOv3 using a rotating hoe. The authors achieved 93.43% mAP in detecting maize, broadleaf weeds, and gramineous weeds, although this precision was not reproduced in the field tests. The robotic system successfully detected and removed the weeds with a weed removal rate of 85.91% and a crop injury rate of 1.17%. However, our study showed higher precision using different models, suggesting that using these ANNs could increase the weed removal rate and reduce the crop injury rate. Additionally, the authors demonstrated how the increase in system speed reduces the accuracy. Therefore, an accurate detection model with reduced inference times would increase the system’s speed without compromising accuracy. Jiang et al. [18] employed a modified YOLOv5 model called SPH-YOLOv5 for an intra-row weeding device based on a vision system and open/close weeding knives. The authors achieved high weed identification mAP (96%) in lettuce cultivation. Our system exceeds this level of accuracy with 97.5% mAP using YOLOv11x in weed and crop identification in maize. Additionally, their system used a movement speed of 3.28 km/h with the SPH-YOLOv5x. Employing an advanced architecture like YOLOv11 may enable the system to perform the task faster, increasing the system’s movement and thus accomplishing weed management in less time. Similarly, Wang et al. [38] used an improved YOLOv5s model for weed identification and spraying in straw-mulched maize fields. The authors distinguished between weeds and crops without identifying the species, achieving 91.4% mAP. Analogously, the YOLOv11s used in our study achieved 91.4% mAP. These results suggest that modifying and adapting the model to the specific task may increase the performance of the results. The improved YOLOv5s model managed to process 76 FPS at a resolution of 640 × 640 pixels, while YOLOv11s approached this value (54 FPS) of processing frames at a higher resolution (1650 × 1650 pixels), which has a major impact on the inference time. Hu et al. [19] proposed an intra-row weed severity classification algorithm for real-time lettuce identification based on an improved YOLOv7l model, which achieved a mAP of 97.1% and could perform 37 FPS. These results are comparable to those reported with YOLOv11x in precision and inference time. Even so, the images used by the authors had a reduced vegetative density. In contrast, our study exceeded 20 instances in several cases, attaining values up to 90 instances per image. The robotic system presented by Balabantaray et al. [37] utilized YOLOv7 for the targeted management of Palmer amaranth in tubs and pots in maize greenhouses. The system detected weeds and crops in densely vegetated images with a precision of 0.732 mAP. Palmer amaranth was successfully detected with an accuracy of 0.909, whereas corn could be detected with a precision of 0.555. In our study, maize was also the most challenging target to detect, yet a precision of 0.863 was achieved with the smallest model (YOLOv11n) and even 0.952 with YOLOv11x. This difference in detection precision may be due to an unbalanced dataset, highlighting the importance of an optimal training set. A distinct robotic system was proposed by Zheng et al. [8], which integrated a YOLOv5s model for accurate identification and localization of cabbage, enabling precise control and dynamic obstacle avoidance for weeding knives. The system was based on detecting only the cabbage plant, permitting the weeding knives to avoid the plant and continue removing weeds in the intra-row zone. The simplicity of detecting only one class enabled the model to achieve accuracies of 96.1% with an inference time of 51 ms. Our study achieved a similar performance in terms of precision, although the weeds and the species of each weed were also detected. This allows species-specific treatment of each weed for more efficient management. In addition, the inference times for a larger image resolution of YOLOv11 performed lower than those achieved by the authors. Even so, the hardware used in our study was superior and ensured greater performance. Inference times would be closer using a similar computing environment and image resolution. However, the results suggest that an advanced model like YOLOv11 could achieve a lower inference time without compromising precision.

Although YOLO algorithms have been the most commonly applied, other models have also been used for weed management. Fan et al. [5] developed a precision weed detection and target spraying system at the seedling stage of cotton fields using an improved Faster R-CNN model. The system achieved a mAP of 98.43% in offline tests and 97.42% in field trials, with an effective spraying rate of 98.93%. Our study obtained a mAP of 91.9%, with Faster R-CNN detecting primarily weeds at the embryonic stage in the inter- and intra-row areas. However, the best result was achieved by YOLOv11 with a mAP of 97.5% in densely vegetated images. In addition, the inference time reported by the authors was 165 ms per frame, while the YOLOv11 model was able to process each frame in 28.91 ms. Indeed, the computational environment marks a difference in this value. A mixed-autonomous robotic system integrating a gantry robot, gripper, and dual RGB-D cameras was developed by Visentin et al. [17] for weed removal in intra- and inter-row zones. The system segmented the crop and weeds, removing the soil background and applying an HSV threshold to the original image. After segmentation, the bounding box of each instance was extracted, creating a new sub-image of each bounding box, which the CNN PlantNet processed to identify the species. The system achieved a promising 98% accuracy in weed detection. Even so, 70% of the crops were successfully identified. However, the robot had to move at 1.8 Km/h to achieve this accuracy. Our study utilizing YOLOv11x achieved 98.2% precision in weed detection and identified the crop with 95.2% accuracy. Using a detection model directly may increase the robot’s speed as detections would be performed more efficiently.

Other authors have developed systems to detect intra-row weeds without employing ANNs, like the tractor-drawn developed by Chandel et al. [6]. They integrated an inter- and intra-row weeding system using rotary tines for intra-row and passive tines for inter-row weeding in maize and pigeon pea fields. Overall weed mortality with the system was 92.8% in maize and 84.1% in pigeon pea, with total plant damage ranging from 4 to 9% in maize and 5 to 8% in pigeon pea crops. Trajkovski et al. [12] proposed a simple modular lightweight e-hoe system for inter- and intra-row weed control for small-scale vegetable producers. Their system achieved a weed removal efficiency of up to 95%, maintaining crop damage below 7%. These mechanical controls achieved promising results in weed removal efficiency and crop damage. However, using ANNs facilitates greater specialization in the field where the system is intended to be used. Using a generalized system for different crop fields may cause variation in accuracy, as shown by Chandel et al. [6], who achieved 92.8% precision in maize, while the accuracy was reduced to 84.1% for pigeon peas. A detection model permits system customization for specific fields aiming to achieve maximum accuracy in its application regardless of the crop.

As highlighted in the study, the intra-row area is challenging to detect and manage weeds. The weeds’ proximity to the crops reduces yields and increases the chances of crop damage during weed treatment [6,8]. Moreover, several of the systems proposed for their treatment performed inter- and intra-row weeding operations separately, demanding large power and operational time [6]. Some advanced solutions are capable of handling both areas, although this equipment can be extremely slow and only effective when the weeds are clearly separated and away from the crop [17]. Our approach addresses these challenges by employing a State-of-the-Art neural network, enabling fast and accurate detections in both inter- and intra-row areas. YOLOv11 could detect the crop and weeds in both areas with precision ranging from 86.7% to 97.5% according to the model size. Regarding growth stages, the dataset comprised images at very early phenological stages, from their first emergence, the most favorable time for successful weed control [39]. Promising results were achieved on weeds at the early stages of development, with many in an embryonic state and thus more difficult to detect and manage, as shown in Figure 9. In addition, the images used in this study showed dense weed vegetation, as shown in Figure 2. On average, each image included 16 labels. Most images (2651) contained 10 to 20 instances, while 781 images had fewer than 10 instances, and 956 images featured between 20 and 92 instances. This dense vegetation added difficulty to the detection process through the models, demonstrating their excellent ability to identify weeds. Furthermore, detection was accomplished with low inference times, facilitating their use in real-time systems. The real-time performance and energy consumption of the different sizes were analyzed. In real-time environments, finding the right balance between accuracy and efficiency is critical due to the hardware limitations and the opportunity to save costs in the system. The high accuracy of YOLOv11 for all sizes enables the model to be adjusted to the hardware requirements, compromising precision or inference speed according to the demands. This variation in model size enables its use in low-power systems, reducing costs by implementing a YOLOv11 size optimized for such hardware to be employed. However, the performance of the models was achieved in theoretical experiments. The detection models were not deployed in a robotic system to treat a field and test their capability in a real environment. Therefore, their accuracy may vary, yet this variation is expected to be minor. In future work, this solution may be deployed in a real system to test its effectiveness. Nevertheless, this approach presents problems with completely occluded weeds as they are not detectable by the model. This may occur when the weeds are situated too close to the crop, where the leaves hide the weeds from the angle where the camera is installed. Therefore, a potential future improvement would be to perform detection using two cameras at different angles to avoid occlusions and increase precision. As a result, this new approach would decrease the speed of the detection process due to the additional computations required for detection in exchange for a gain in accuracy, thus potentially bringing excellent detection results to sizes such as YOLOv11n. Despite some limitations, the approach proposed in this study may be used for different crop fields. This research tested the performance in maize fields, but the detection models can be trained and specialized for different crop fields. Consequently, the technique used to detect intra-row weeds can be specifically designed for the particular crop field where the weed treatment is required.

## 5. Conclusions

Weeds in inter- and intra-row zones continue to undermine agricultural productivity, with intra-row weeds being particularly difficult to manage due to their proximity to crops and high occlusion rates. This study explored the potential of advanced DL models for automated weed detection, evaluating their accuracy, hardware performance, and suitability for real-world applications. YOLOv11 demonstrated exceptional capabilities among the tested models, achieving a mAP of 97.5% and real-time processing at 34 frames per second. YOLOv11m was identified as the optimal model variant for field deployment, offering a balance between precision (mAP 94.4%) and resource efficiency. RT-DETR also delivered competitive performance, achieving a mAP of 97.2% and a respectable inference speed of 27 frames per second. Conversely, Faster R-CNN showed a solid but comparatively lower performance, with a mAP of 91.9% and significantly slower inference times (11 frames per second), making it less suitable for real-time applications. The proposed system effectively addressed the complexities of intra-row weed detection and shows promising potential for integration into robotic platforms. Future research should focus on field implementation, including live testing in real-world agricultural systems. Further enhancements, such as multi-angle detection systems, could reduce occlusion errors and enhance overall performance. By leveraging advanced detection models, this approach paves the way for sustainable weed management practices, improving crop yields while reducing reliance on chemical herbicides and manual labor.

## Figures and Tables

**Table 1:** Number of images and annotations for each crop class and weed species within each dataset split.

	Training	Validation	Testing	Total
Number of Images	3071	438	879	4388
ZEAMX	13,128	1864	3785	18,777
SOLNI	9374	1307	2753	13,434
CYPRO	10,693	1485	3287	15,465
ECHCG	4674	630	1388	6692

**Table 2:** Training hyperparameters for Faster R-CNN, RT-DETR, and YOLOv11.

Hyperparameters	Values
Epochs	300
Patience	25
Optimizer	AdamW
Learning rate	0.001
Image size	1650
Batch	4
Number of classes	4
Test IoU	0.5
Test confidence	0.4

**Table 3:** Comparison performance of the different detection architectures.

Model	Faster R-CNN	RT-DETR	YOLOv11
ZEAMX	0.868	0.947	0.952
SOLNI	0.95	0.988	0.987
CYPRO	0.915	0.971	0.974
ECHCG	0.943	0.983	0.985
**mAP**	0.919	0.972	0.975

**Table 4:** Hardware performance comparison of the employed detection architectures.

Model	Faster R-CNN	RT-DETR	YOLOv11
Parameters (M)	59.99	67.3	56.9
Model size (MB)	126.47	133.03	114.01
Training VRAM (GB)	24.7	29.2	30.4
FLOPS (G)	594	232.4	195.5
Inference time (ms)	90.37	35.92	28.91
Inference FPS	11	27	34

**Table 5:** Detection performance of the different sizes of YOLOv11.

Model	YOLOv11x	YOLOv11l	YOLOv11m	YOLOv11s	YOLOv11n
ZEAMX	0.952	0.916	0.897	0.871	0.863
SOLNI	0.987	0.97	0.964	0.929	0.842
CYPRO	0.974	0.955	0.945	0.904	0.85
ECHCG	0.985	0.975	0.969	0.95	0.912
**mAP**	0.975	0.954	0.944	0.914	0.867

**Table 6:** Comparison of hardware performance across the utilized model sizes.

Model	YOLOv11x	YOLOv11l	YOLOv11m	YOLOv11s	YOLOv11n
Parameters (M)	56.9	25.3	20.1	9.4	2.6
Model size (MB)	114.01	53.80	42.82	22.59	9.10
Training VRAM (GB)	30.4	20.4	15.8	9.1	5.2
FLOPS (G)	195.5	87.3	68.2	21.6	6.4
Inference time (ms)	28.91	24.86	20.92	18.39	16.97
Inference FPS	34	40	47	54	58

## Data Availability

The datasets presented in this article are not readily available because the data are part of an ongoing study.
